# Novel Parvovirus Related to Primate Bufaviruses in Dogs

**DOI:** 10.3201/eid2406.171965

**Published:** 2018-06

**Authors:** Vito Martella, Gianvito Lanave, Eszter Mihalov-Kovács, Szilvia Marton, Renáta Varga-Kugler, Eszter Kaszab, Barbara Di Martino, Michele Camero, Nicola Decaro, Canio Buonavoglia, Krisztián Bányai

**Affiliations:** Autor affiliations: University of Bari, Bari, Italy (V. Martella, G. Lanave, M. Camero, N. Decaro, C. Buonavoglia);; Hungarian Academy of Sciences, Budapest, Hungary (E. Mihalov-Kovács, S. Marton, R. Varga-Kugler, E. Kaszab, K. Bányai);; University of Teramo, Teramo, Italy (B. Di Martino)

**Keywords:** dog, parvovirus, protoparvovirus, bufavirus, CIRD, kennel cough, viruses, respiratory infections, enteric infections

## Abstract

A novel protoparvovirus species, related genetically to human bufaviruses, was identified in dogs with respiratory signs. The canine bufavirus was distantly related to the well-known canine protoparvovirus, canine parvovirus type 2, sharing low amino acid identities in the nonstructural protein 1 (40.6%) and in the capsid protein 1 (33.4%). By screening collections of fecal, nasal, and oropharyngeal samples obtained from juvenile dogs (<1 year of age), canine bufavirus DNA appeared as a common component of canine virome. The virus was common in the stool samples of dogs with or without enteric disease and in the nasal and oropharyngeal swab samples of dogs with respiratory signs. However, the virus was not detected in nasal and oropharyngeal swab samples from animals without clinical signs.

Parvoviruses (family *Parvoviridae*) are small, nonenveloped viruses of 25–30 nm in diameter, with an icosahedral capsid. The genome is a single-strand DNA of 4.5–5.5 kb (*1*) with complex hairpin structures at the 5′ and 3′ ends. The genome is predicted to encode 3 or 4 proteins: nonstructural (NS) 1, nucleoprotein (NP) 1, and viral protein (VP) 1 and 2.

Parvoviruses have long been known in dogs, since the identification of canine minute virus, or canine parvovirus (CPV) type 1 (CPV-1; genus *Bocaparvovirus*), in 1967 from the fecal samples of healthy dogs (*2*). CPV-1 infection is responsible for reproductive disorders and occasionally for respiratory and gastrointestinal signs in young puppies (*3*). A second CPV (CPV-2; genus *Protoparvovirus*) was reported in the 1970s in Europe and North America in puppies with signs of hemorrhagic gastroenteritis and myocarditis (*4*). CPV-2 is currently regarded as the major causative agent of severe gastroenteritis in puppies and is included in canine core vaccination schedules globally (*5*). In 2011, a second canine bocaparvovirus (CBoV) was identified from nasal swabs of healthy and sick dogs (*6*); a third species of CBoV was identified in 2013 in the liver of a dog with multiorgan failure (*7*) (Table). Whether the newly identified parvoviruses play a role as canine pathogens has not yet been assessed.

**Table:** Parvoviruses identified in dogs and their classification and proposed classification of canine bufaviruses*

Genus and species	Common/used names in literature	Year identified	Place identified	Reference	GenBank accession no.
*Bocaparvovirus*					
Carnivore bocaparvovirus 1	CPV-1, minute virus of canines (MVC) or CBoV-1	1970	United States	(*2*)	FJ214110
Carnivore bocaparvovirus 2	CBoV-1 or CBoV-2	2011	United States	(*6*)	JN648103
Carnivore bocaparvovirus 3	Feline bocaparvovirus	2009	United States	(*8*)	JQ692585
Carnivore bocaparvovirus 4†	CBoV-3	2011	United States	(*7*)	KC580640
*Protoparvovirus*					
Carnivore protoparvovirus 1	CPV-2	1978	United States	(*4*)	
	CPV-2a	1983	United States	(*9*)	M24000
	CPV-2b	1984	United States	(*10*)	M74849
	CPV-2c	2000	Italy	(*11*)	AY380577
Carnivore protoparvovirus 2†	Canine bufavirus	2012–2016	Italy and Hungary	This study	MF198244–46

*CBoV, canine bocavirus; CPV, canine parvovirus. †Candidate novel species.

We report the identification of a novel CPV. We determined the genome sequence of the CPV and designed specific primers and probes useful for laboratory diagnosis. Screening of enteric and respiratory samples from dogs with either gastroenteric or respiratory disease and from animals without clinical signs suggested a possible association between the novel virus and respiratory disease in young dogs.

## Material and Methods

### Identification of the DNA of a Novel Parvovirus

In 2011, an outbreak of canine infectious respiratory disease (CIRD) occurred in a litter of 3 mixed-breed 5-month-old puppies in Italy. The animals’ clinical signs were nasal discharge, coughing, and respiratory distress, but they completely recovered from the disease after 2 weeks. Nasal and oropharyngeal swab specimens tested negative to a panel of molecular assays for CIRD-associated common and emerging viral agents: canine adenovirus (CAV) types 1 and 2, canine distemper virus, canid herpesvirus 1 (CHV-1), canine respiratory coronavirus, influenza virus, canine parainfluenza virus, canine pneumovirus, nonprimate canine hepacivirus, *B. bronchiseptica*, *Streptococcus equi* subspecies *zooepidemicus*, and *Mycoplasma cynos* (*12*). Because the etiology of the outbreak was unknown, the case was considered eligible for metagenomic investigation. 

We performed random primed reverse transcription PCR and PCR assays on pooled samples (the nasal and oropharyngeal swab specimens of the 3 puppies) to amplify nucleic acids and used them as templates for next-generation sequencing (NGS) experiments on the Ion Torrent platform (New England Biolabs, Ipswich, MA, USA) (*13*). We evaluated sequence data using CLC Genomic Workbench (http://www.clcbio.com). NGS revealed the presence of parvovirus-related sequence reads that mapped to human bufaviruses. We mapped a total of 3,530 reads to human bufavirus and assembled them into 3 contigs, 422-, 416-, and 191-nt long. We reconstructed the nearly complete genomic sequence of the new CPV (canine bufavirus [CBuV]), strain ITA/2011/297-15, by combining 5′ rapid amplification of cDNA ends (RACE) protocols (*14*) with minor modifications, using the kit 5′ RACE System for Rapid Amplification of cDNA Ends version 2.0 (Life Technologies, Paisley, UK) and a primer-walking strategy with specific primers designed to close the gaps among noncontiguous sequences. We purified and cloned the amplicons using a TOPO XL Cloning Kit (Life Technologies) and generated consensus sequences by sequencing >3 clones for each PCR fragment.

### Screening of Samples in Conventional and Quantitative PCR

We designed specific primers on the VP2 genomic region of strain ITA/2011/297-15 for quantitative detection in real-time PCR (qPCR) (CPPV-L3-for 5′ TGAACAAGAAATAGACAACATTGTCAT 3′, CPPV-L3-rev 5′ AAAGAGCAGTTAGGTCATTGTTGT 3′, and CPPV-L3 Pb 5′ Fam CCAAACAAGGTACAGGACAGGAAGAAACAACACAA BHQ1 3′). We calculated CBuV DNA copy numbers on the basis of standard curves generated by 10-fold dilutions of a plasmid standard TOPO XL PCR containing a 500-nt fragment of the VP2 region of strain ITA/2011/297-15. We added 10 μL of sample DNA or plasmid standard to the 15-μL reaction master mix (IQ Supermix; Bio-Rad Laboratories SRL, Segrate, Italy) containing 0.6 μmol/L of each primer and 0.2 μmol/L of probe. Thermal cycling consisted of activation of iTaq DNA polymerase at 95°C for 3 min, 42 cycles of denaturation at 95°C for 10 s, and annealing extension at 60°C for 30 s. We evaluated the specificity of the assay with a panel of canine DNA viruses (CPV-1, CPV-2, CHV-1, circovirus, CAV-1, and CAV-2). The qPCR detected >10^1^ DNA copies/10 μL of standard DNA and 8.78 × 10^0^ DNA copies/10 μL of DNA template extracted from clinical samples. CBuV quantification had an acceptable level of repeatability over various magnitudes of target DNA concentrations, when calculating (*15*) the intraassay and interassay coefficients of variation within and between runs, respectively.

In addition, we designed specific primers (CPPV 165F 5′ CTGGTTTAATCCAGCAGACT 3′ and CPPV 371R 5′ TGAAGACCAAGGTAGTAGGT 3′) to amplify and sequence a 207-nt fragment of VP2. We used the AccuPrime Taq DNA polymerase (Life Technologies) for PCR amplification. Cycling thermal conditions included initial activation of the polymerase at 94°C for 2 min, 45 cycles at 94°C for 30 s, 53°C for 30 s, and 72°C for 30 s, followed by final extension at 72°C for 10 min.

### Respiratory Samples

During 2011–2015, we obtained nasal and oropharyngeal swab (NOP) samples from 58 pups and young dogs (<1 year of age) with CIRD (collection RIS); the animals were early acute clinically ill CIRD dogs with onset of respiratory signs at 0–3 days at the time of sample collection. The RIS samples were a subset of a larger collection used for surveillance of traditional and emerging pathogens associated with CIRD (*12*). We collected samples from the nasal and oral cavities in parallel and stored them separately. We also screened NOP swabs obtained from 90 dogs <1 year of age without clinical signs (collection RIA) as controls. We collected all samples with dry swabs and immediately stored them at −20°C.

### Enteric Samples

We screened archived enteric samples (stools and rectal swabs) collected at the Department of Veterinary Medicine, University of Bari, Italy, during 2010–2015 for CBuV. The samples had been obtained from pups and young dogs (<1 year of age). We screened 81 samples from animals with signs of gastroenteritis (collection EIS) and 78 samples of animals without clinical signs (collection EIA).

We also searched for CBuV DNA in a collection of enteric samples from pups and young dogs (<1 year of age) either with gastroenteritis (collection EHS) or without clinical signs of gastroenteritis (collection EHA), obtained in Hungary in 2012 and available at the Institute of Veterinary Medical Research, Hungarian Academy of Science, Budapest. We screened 40 samples from healthy animals and 20 samples from animals with clinical signs.

### Statistical Analysis

We evaluated the associations among clinical signs, age, and presence of the virus in the respiratory and enteric samples by the χ^2^ test using a web-based software program (R version 3.3.0; http://www.r-project.org/). We set statistical significance to p<0.05.

### Genome Analysis of Hungarian CBuV Strains

We selected samples positive for CBuV on the basis of the genome copies as revealed by quantitative PCR. We also identified the genomes of 2 additional CBuV strains, HUN/2012/22 and HUN/2012/126.

### Sequence and Phylogenetic Analyses

We retrieved genome sequences of protoparvovirus strains from GenBank and aligned them using the Clustal Omega tool from the European Molecular Biology Laboratory (https://www.ebi.ac.uk/Tools/msa/clustalo/). We conducted sequence and phylogenetic analyses in Geneious version 9.1.8 (Biomatters Ltd., Auckland, New Zealand); we used the neighbor-joining method, Jukes–Cantor genetic distance model, and bootstrapping for <1,000 replicates.

### Virus Cultivation

We homogenized PCR-positive respiratory and fecal samples in 10% Dulbecco’s modified Eagle’s medium and then centrifuged them at 10,000 × *g*. We filtered the supernatant with 0.22-μm filters and inoculated it onto freshly seeded canine fibroblastic tumor (A-72) cells, incubated at 37°C in 5% CO_2_. We also inoculated Madin-Darby canine kidney cell (MDCK) and Walter Reed canine cell (WRCC) lines. We evaluated viral growth through 5 serial passages, monitoring the onset of cellular cytopathic effect and testing the cell supernatant by qPCR.

## Results

### Genome Analysis of Canine Bufaviruses

**Figure 1:**
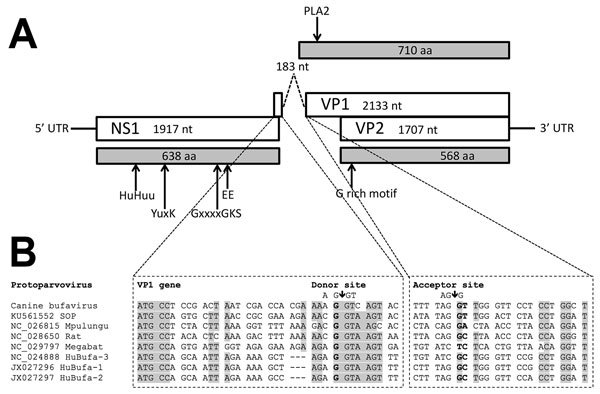
Genome organization of canine bufavirus. A) Positions of the conserved helicase Walker A (GxxxxGKS), Walker B (EE), and replication initiator motifs (HuHuu and YuxK) in NS1 and of the phospholipase A2 (PLA2) and glycine-rich region (G-rich) in VP1 and VP2. B) Putative splicing mechanism in the VP1 gene of canine bufavirus, human bufaviruses, and other protoparvoviruses. Two potential splice sites are a potential donor site (AG↓GT) at nt 1931 and an acceptor site (AG↓G) at nt 2115. The putative VP1 sequence starts with ATG at the end of ORF1 at nt 1906 upstream of the splice donor site at nt 1931. Gray shading indicates strictly and highly conserved bases. GenBank accession numbers are provided for reference sequences. NS, nonstructural; UTR, untranslated region; VP, viral capsid protein.

We determined the nearly complete genome (4,537 nt) of the CBuV strain ITA/2011/297-15, including a partial 5′ untranslated region (UTR) (310 nt), the complete NS1 sequence (638 aa), the complete VP1 (710 aa) and VP2 (568 aa) sequences, and a partial 3′ UTR (8 nt). The genome coding sequence, excluding the terminal UTR regions, was 4,219 nt (GenBank accession no. MF198244). The genome contained 2 major open reading frames (ORFs); the left ORF, coding for NS1, was 1,917 nt and the right ORF, encoding VP1 and VP2, was 2,316 nt (Figure 1, panel A). Full-genome sequence alignment showed a high degree of sequence divergence, up to 58% overall nucleotide identity to most parvoviruses but not bufaviruses. CBuV was more closely related to bufaviruses identified in primates (61.6%–63.2% nt similarity), pigs (59.6% nt), and bats (58% nt) (*16**–**20*) and more distantly related to CPV-2 (45% nt) (Technical Appendix Table). The putative bufavirus NS1 start codon was located in a strong Kozak sequence, ACCATGG. The ATP- or GTP- binding Walker loop motif (GXXXXGK[T/S]) was found in NS1 (405-GPASTGKS-412) (*21*). In addition, the NS1 contained 2 conserved replication initiator motifs, GL**H**F**H**VLLQ and IVR**Y**FLTKQP (boldface type indicates conserved amino acids) (*22*). We generated an nt and aa sequence identity matrix. NS1 showed <69.4% nt and 51.4% aa identity with other parvovirus NS1 sequences, including its closest relatives in the *Parvovirus* genus (Technical Appendix Table).

The termination of ORF1 overlapped the start of ORF2 by 14 nt. From sequence alignment and comparison with other BuVs, we detected 2 potential splice sites in the ORF1/ORF2 junction, a potential donor site (AG↓GT) at nt 1931 and an acceptor site (AG↓G) at nt 2115. The putative VP1 sequence started at the end of ORF1 at nt 1906, upstream of the splice donor site at nt 1931 (Figure 1, panel B). We found the phospholipase A2 (PLA2) motif (Figure 1, panel A), with its highly conserved calcium binding site (YLGPG), in the main ORF of VP1. The phospholipase catalytic residues (HD and D) were present at amino acid positions 41–42 and 63 (Figure 1, panel A). The VP1 showed <67.2% aa identity to other genera of the family *Parvovirinae*, including its closest relative in the *Parvovirus* genus (Technical Appendix). The N terminus of the CBuV VP2 protein contained a glycine-rich sequence (GGGGGGGSGVG) that was also present in other parvoviral VP2 proteins (Figure 1, panel A).

We successfully determined the complete coding genome sequence of 2 additional CBuV strains of enteric origin, HUN/2012/22 (GenBank accession no. MF198245) and HUN/2012/126 (GenBank accession no. MF198246); the sequence was 4,219 nt (4,463 and 4,308 nt with the partial UTRs, respectively). Overall, the 3 CBuV strains displayed 99.8%–99.9% nt identity to each other, with only 1 nonsilent mutation in the NS1 protein and 1 nonsilent mutation in the VP2 protein.

**Figure 2:**
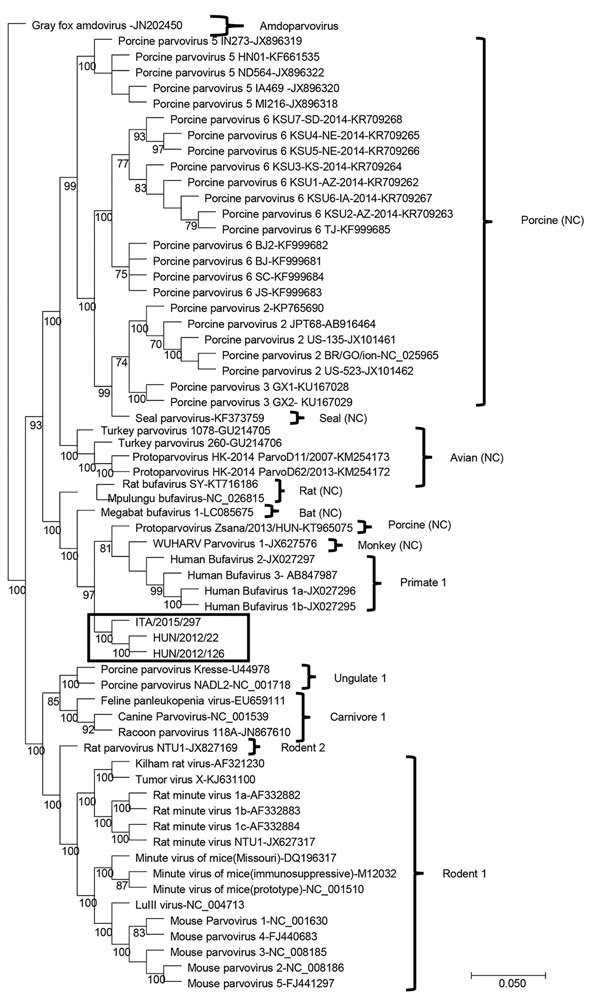
Capsid-based phylogenetic tree displaying the diversity of protoparvoviruses. The protoparvoviruses officially recognized by the International Committee on Taxonomy of Viruses are included, along with nonclassified (NC) protoparvoviruses. The tree was generated using the neighbor-joining method with the Jukes-Cantor algorithm of distance correction, with bootstrapping over 1,000 replicates. Box indicates canine bufavirus strains. GenBank accession numbers are provided for reference isolates; gray fox amdovirus (GenBank accession no. JN202450) is used as outgroup. Scale bar indicates nucleotide substitutions per site.

Upon phylogenetic analysis (Figure 2), the CBuV strains segregated into a well-defined group (bootstrap value 100), encompassing parvoviruses identified in rats, bats, pigs, and primates. The closest relatives within this group were parvoviruses from humans, commonly called human bufaviruses (*16**,**17*), and parvoviruses detected in monkeys (*Macaca mulatta*) (*18*) and pigs (*19*).

### Screening in PCR of Canine Samples

#### Respiratory Samples

Molecular screening by qPCR detected CBuV DNA in 10/58 (17.2%) oropharyngeal swabs and 15/58 (25.8%) nasal swabs from collection RIS. In total, 31% (18/58) of the animals tested positive for CBuV in either the pharyngeal or nasal sample, whereas 12.1% (7/58) were positive in both the oral and nasal swab sample. We did not detect CBuV DNA in the NOP samples of the control group (0/90) (collection RIA). The viral loads of collection RIS ranged from 4.91 × 10^1^ to 8.78 × 10^3^ DNA copies/10 μL of template (mean 8.73 × 10^2^ DNA copies; median 2.91 × 10^2^ DNA copies). We found a statistically significant difference for CBuV prevalence in oropharyngeal (odds ratio [OR] 6, 95% CI 1.6–23) and nasal (OR 10.1, 95% CI 2.7–36.8) swab specimens when we compared animals with and without clinical signs (p<0.05 for both comparisons).

We reanalyzed the results by age-based cohorts of animals (0–6 and 7–12 months). In the 0–6-month group, only 1/17 (5.8%) animals were positive; 17/41 (41.4%) tested positive in the 7–12-month group. However, this difference was not statistically significant (p>0.05).

We also screened the NOP swabs of the litter infected by strain ITA/2011/297-15 in qPCR. We detected CBuV DNA in all 3 puppies, with viral titers as high as 2.79 × 10^2^, 1.01 × 10^3^ DNA, and 3.77 × 10^3^ copies/10 μL of template.

#### Enteric Samples

Molecular screening by qPCR revealed CBuV DNA in 26/81 (32.1%) stools or rectal swabs from collection EIS and 15/78 (19.2%) samples of collection EIA. The viral loads of collection EIS ranged from 4.91 × 10^1^ to 8.78 × 10^3^ DNA copies/10 μL of template (mean 4.21 × 10^2^ DNA copies; median 1.38 × 10^2^ DNA copies). The viral loads of collection EIA ranged from 5.10 × 10^1^ to 4.34 × 10^2^ DNA copies per 10 μL of template (mean 1.76 × 10^2^ DNA copies; median 1.48 × 10^2^ DNA copies). We found no statistically significant difference between enteric signs and the presence of CBuV DNA in stools or rectal swabs.

We detected CBuV DNA in 8/20 (40%) enteric samples from collection EHS and 19/40 (47.5%) samples from collection EHA. The viral loads of collection EHS ranged from 1.31 × 10^1^ to 5.42 × 10^3^ DNA copies/10 μL of template (mean 6.55 × 10^2^ DNA copies; median 3.99 × 10^1^ DNA copies). The viral loads of collection EHA ranged from 1.21 × 10^1^ to 2.57 × 10^10^ DNA copies/10 μL of template (mean 1.35 × 10^9^ DNA copies; median 2.92 × 10^2^ DNA copies). We found no statistically significant difference between enteric signs and the presence of CBuV DNA in the samples from Hungary.

We subjected samples containing genome copies >10^3^ DNA copies/10 μL of template to PCR amplification with primers CPPV 165F and CPPV 371R, which amplify a 207-nt fragment of VP2. We successfully sequenced 12 samples, yielding amplicons of the expected size in PCR and confirming the specificity of the qPCR.

#### Virus Cultivation

We visually inspected the inoculated monolayers of A-72, MDCK, and WRCC cells after 5 serial passages and monitored virus titer in cellular supernatant by qPCR. We did not observe viral growth in any of the cells. 

## Discussion

We report the detection and genomic characterization of CBuV, a novel canine protoparvovirus, from a small outbreak of CIRD. In the NS1 gene, CBuV displayed low nt (24.1%–69.4%) and aa (19.3%–51.4%) identities compared with other protoparvoviruses. Current International Committee on Taxonomy of Viruses criteria for classification of parvoviruses into the same species require >85% aa identity in the NS1 protein; on this basis, CBuV could be classified as a new parvovirus species. The closest relatives to CBuV were protoparvoviruses identified in primates and other mammals (*16*–*19*), commonly termed as bufaviruses. Bufaviruses were first identified in 2012 in Burkina Faso in fecal samples from a child with enteric signs (*16*). Similar bufaviruses have been subsequently identified in different species of domestic and wild animals (*18**–**20*). CBuV exhibited the most similarity in NS1 (66.9%–69.4% nt and 47.2%–51.4% aa) and VP1 (66%–68.2% nt and 62.5%–67.2% aa) to bufaviruses identified in primates. In addition, the genetic organization of CBuV resembled that of primate bufaviruses, with the conservation of a potential splicing mechanism regulating VP1 translation. On the other hand, CBuV was genetically more distantly related to CPV-2, showing only 56% nt and 40.6% aa identity in the NS1 and 42.6% nt and 33.4% aa identity in the VP1 between the 2 canine protoparvoviruses. Because we were not able to adapt the virus to in vitro growth in different canine cell lines, we could not assess whether there is an antigenic relationship between the 2 canine protoparvoviruses, CBuV and CPV-2, in cross-neutralization studies.

A small case–control study on samples of enteric and respiratory origin in puppies and young dogs (<1 year of age) revealed that CBuV appeared significantly more common in NOP swab samples from dogs with acute CIRD. We detected CBuV DNA in 17.2% (10/58) of oropharyngeal swab specimens and 25.8% (15/58) of nasal swab specimens from collection RIS. In total, 31% (18/58) of the animals with CIRD tested positive for CBuV, whereas the virus was not detectable in respiratory samples of 90 animals without clinical signs. When we analyzed the results of collection RIS by age-based cohorts, we observed an increased prevalence (41.4%, 17/41) in the 7- to 12-month age group, whereas only 1/17 animals (5.8%) in the 0- to 6-month age group tested positive for CBuV. We also found a high prevalence of CBuV DNA in canine stool samples, although we observed no substantial difference between dogs with enteric disease and clinically healthy dogs. These findings indicate that CBuVs are a common component of the canine fecal virome.

With certain exceptions, it has been difficult to demonstrate a clear association of many potential pathogens with CIRD in either epidemiologic studies or experimental infections. Our study could not provide conclusive evidence for a role of this novel virus in CIRD.

CIRD or kennel cough has a multiagent etiology, with >1 agent (viruses or bacteria) involved sequentially or synergistically to cause disease (*23*). Pathogens commonly associated with CIRD include CAV-2, canine parainfluenza virus, and *Bordetella bronchiseptica* (*12*,*24*,*25*). Less commonly, CHV-1 can cause respiratory disease (*26*). CAV-1 and canine distemper virus infections are also associated with respiratory disease but are usually responsible for systemic disease (*26*,*27*).

In recent years, other emerging agents have been associated with CIRD, including canine respiratory coronavirus (*27*,*28*), canine pneumovirus (*29*), nonprimate canine hepacivirus (*30*), CBoVs (*6*), *Mycoplasma cynos* (*31*), and *Streptococcus equi* subsp. *zooepidemicus* (*32*,*33*). In addition, thus far, >5 strains of influenza virus have been identified in dogs: the equine-derived H3N8 virus, the human-derived H1N1 virus, and the avian-like H3N2, H3N1, and H5N2 viruses (*34*,*35*).

Of interest, the titer of CBuV in the NOP samples was not high, with a median value of 2.91 × 10^2^ DNA copies/10 μL. This low level of virus shedding in NOP secretions was difficult to interpret, and intrinsic properties of the virus, or the dynamics of virus shedding at the time of sampling, or both could account for it. For comparison, a human parvovirus associated with respiratory disease, human bocaparvovirus 1, can be shed at titers as high as 3.9 × 10^11^ copies/mL in NOP samples (*36*). It is worth noting that we also found high loads of CBuV (<2.57 × 10^10^ DNA copies/10 μL of template) in 4 fecal samples that substantially exceeded the median value (2.92 × 10^2^ DNA copies/10 μL) of collection EHA.

To date, scientists have searched for bufaviruses almost exclusively in fecal samples and have detected them in diarrheal stools of patients of all ages worldwide. The prevalence of bufaviruses in human patients ranges from 0.3% to 4%, and their etiologic role in enteric or extraenteric diseases remains uncertain (*16*,*17*,*37**–**41*). Bufaviruses have also been found in other mammalian hosts, including wild and captive nonhuman primates, swine, shrews, rats, bats, and fur seals (*18**–**20*,*42**–**46*). Of interest, bufaviruses have also been detected in the serum and spleens of monkeys and in the spleens of shrews (*18*,*44*) and in a unique NOP sample of 955 human patients with lower respiratory tract signs (*47*), suggesting the possibility of extraintestinal or systemic infections. In this study, we have also reconstructed the genome sequence of 2 CBuV strains detected in canine fecal samples. By genome comparison, we observed only 2 aa differences between the CBuV strains of respiratory and enteric origin, although the viruses were identified from animals of different geographic origin (i.e., Italy and Hungary). Although our findings corroborate earlier evidence that bufaviruses can target extraintestinal tissues and organs, only animal experiments or detailed observational studies can fully address this issue.

In conclusion, the advancement of techniques available for pathogen discovery is quickly broadening the list of potential canine infectious agents. Understanding in more depth the effects of those agents on canine health will be pivotal to implementing future strategies for prophylaxis, chiefly for complex diseases like CIRD.

Technical Appendix Additional information about canine bufaviruses and other parvoviruses.
