# Supplementary material for: Novel Parvovirus Related to Primate Bufaviruses in Dogs
Source: Emerg Infect Dis. 2018 Jun;24(6):1061–8. doi: 10.3201/eid2406.171965 (PMC6004837; doi:10.3201/eid2406.171965)
Supplement: Technical Appendix — Additional information about canine bufaviruses and other parvoviruses. [file 17-1965-Techapp-s1.pdf]

Article DOI: https://doi.org/10.3201/eid2406.171965

Novel Parvovirus Related to Primate Bufaviruses in Dogs

Technical Appendix

Technical Appendix Table. Sequence identity percentages between canine bufaviruses and other parvoviruses in the complete genome and in selected proteins*

	ITA/2011/297–15					HUN/2012/22					HUN/2012/126				
	Gen	NS1		VP1		Gen	NS1		VP1		Gen	NS1		Capsid	
Virus†	nt	nt	aa	nt	aa	nt	nt	aa	nt	aa	nt	nt	aa	nt	aa
Canine bufaviruses															
Ca/ITA/2011/297–15	/	/	/	/	/	99.8	99.8	99.6	99.9	99.6	99.8	99.8	99.7	99.9	99.6
Ca/HUN/2012/22	99.8	99.8	99.6	99.9	99.6	/	/	/	/	/	99.9	99.9	99.8	99.9	99.8
Ca/HUN/2012/126	99.8	99.8	99.7	99.9	99.6	99.9	99.9	99.8	99.9	99.8	/	/	/	/	/
Other parvoviruses															
Hu/BuV 1a/JX027296	61.6	67	47.5	68.2	64.5	61.7	67	47.6	68.1	64.6	61.7	67	47.6	68.1	64.7
Hu/BuV 1b/JX027295	61.7	67	47.2	68.3	64.5	61.7	67	47.3	68.2	64.6	61.7	67.1	47.3	68.2	64.7
Hu/BuV 2/JX027297	61	66.9	47.9	66	62.5	61	66.9	48	66	62.7	61	67	48	66	62.9
Hu/BuV 3/AB847987	61.1	67.1	48.4	66.6	62.8	61.1	67.2	48.4	66.5	62.9	61.1	67.3	48.5	66.5	63
Si/WUHARV/JX627576	63.2	**69.4**	51.4	**67.9**	**67.2**	63.2	**69.4**	51.4	**67.8**	**67.4**	63.3	**69.5**	51.4	**67.8**	**67.6**
Po/Zsana/2013/HUN/KT965075	59.6	65.7	46.5	65.9	65.3	59.6	65.7	46.5	66	65.8	59.6	65.7	46.5	66	66
Bat/Megabat BuV 1/LC085675	58	65.3	45.7	61.1	60.7	59.4	65.2	45.7	61.1	60.9	59.4	65.2	45.7	61.1	61
Ro/Mpulungu BuV/NC_026815	57	64.7	44.9	55.9	54.9	57	64.3	45	55.8	55.1	57	64.4	45	55.8	55.1
Ro/Rat BuV SY/KT716186	56.7	63.7	43.1	55	54.1	56.8	63.2	43.2	54.9	54.5	56.8	63.3	43.2	54.9	54.5
Ca/CPV-2/NC_001539	45	56	40.6	42.6	33.4	45	56	40.6	42.6	33.2	45	56.1	40.7	42.6	33.2

*Gen, genome; NS, nonstructural protein; VP, virus capsid protein. Bold type indicates highest identities.
†Ca, canine; Hu, human; Po, porcine; Ro, rodent; Si, simian.
